# Editorial: Neuronal Plasticity and Neuromodulation in Development and Developmental Disorders

**DOI:** 10.3389/fneur.2022.912046

**Published:** 2022-05-23

**Authors:** Jean-Pierre Lin

**Affiliations:** ^1^Children's Neurosciences Evelina London Children's Hospital, Guy's and St Thomas' NHS Foundation Trust, London, United Kingdom; ^2^Faculty of Life Sciences and Medicine, Department of Women and Children's Health, School of Life Course and Population Sciences, King's College London, London, United Kingdom

**Keywords:** neuromodulation, developmental neurobiology, neuroplasticity, childhood, neurology

The growth and function of the developing brain are a triumph of hope over expectation against impossible natural odds. Development and pathophysiology compete with each other to achieve rewarding functions and the seemingly endlessly adaptable nervous system is edited and pruned accordingly within windows of neuroplasticity which open and close according to genetic rules and neuro-environmental experiences leading to adaptive complexity, vulnerability and disruption (1). Unsurprisingly, every experience, thought, action and reaction have a *neuromodulating* effect on the developing and developed nervous system. Robust systems survive to become stronger and more adaptive.

As we move from syndromic diagnoses composed of symptoms and signs to fundamentally molecular diagnostic formulations, it is clear that old divisions of neurology into disorders brain, spinal cord and neuromuscular functions dissolve into more basic units of cellular functional behavior and systems neuromodulation.

Diagnosis, the process of discovering the nature of a problem or illness through examination, imaging, neurophysiological, biochemical and genetic testing and prognosis, our understanding of the likely future outcome or course of a diagnosis, will always lag behind this curve of new discoveries and understanding of developing, disordered, brain function.

Neuromodulation seeks to describe and understand dynamic, self-adapting, functional and dysfunctional systems for which dynamic solutions are required.

Clinicians, like philosophers are capable of teaching and defending doctrines, which in theoretical and practical terms obstruct clinical progress. The “fallacy of misplaced concreteness” was coined by the philosopher Alfred North Whitehead. One commits the fallacy of misplaced concreteness when one mistakes an abstract belief, opinion, or concept about the way things are for a physical or “concrete” reality: "There is an error; but it is merely the accidental error of mistaking the abstract for the concrete.” “*Science and the Modern World,” Alfred North Whitehead (1925/1953)*.

In pediatric neurology the attribution of motor dysfunction to disturbances of the corticospinal tract known as the “upper motorneuron” syndrome has long dominated clinical thinking. However in this volume on neuroplasticity, the efficacy of Constraint-Induced Movement Therapy (CIMT) and Bimanual training is reportedly “independent of CST connectivity pattern,” contrary to the study hypothesis of the investigators. Furthermore, “children with an ipsilateral CST lesion, previously thought to be maladaptive, have the capacity to improve as well as children with a contralateral or bilateral CST lesions following intensive CIMT or Bimanual training,” thus overturning a long-held “*misplaced concreteness.”* It is regrettable that this work may take decades to reach undergraduate and junior clinical education and training on the grounds that it is “too specialized” or only applies to pediatrics. But I remember well failing to understand how the upper motor neurone concept alone explained the phenomenology of adult and childhood strokes. Accordingly, and perhaps unsurprisingly the authors report in their concluding remarks that disordered sensorimotor integration may play a significant role in motor dysfunction and this is backed up by reports of greater responses to CIMT in children with poor sensory function (Friel et al.).

Opportunistic study is often required to inform our conceptual understanding of neurodevelopmental processes but require specific clinical conditions such as those which obtained in the measurement of age-related lateralization of cerebral inhibition i.e., “Go” and “No-Go” inhibition which was measured in children undergoing continuous subdural electrocorticography (ECoG) recordings for epilepsy surgery localization. This revealed a predominantly right-sided inhibition in the inferior frontal gyrus (IFG) associated with theta (4–8 Hz) and high-gamma (HG) (70–200 Hz) power spectra, thus giving us an opportunistically-derived developmental insight into such inhibitory functions (Kuo et al.).

At the opposite end of the spectrum, mathematical modeling of chronic deep brain stimulation (DBS)-dependency i.e., susceptibility to *status dystonicus* following abrupt withdrawal of continuous DBS neuromodulation for intractable dystonia, is revealed as highly dependent on the “neuroplastic nature of a disorder” or an individual. This could help modeling predicted responses to DBS withdrawal. This model predicts that insertion and withdrawal of DBS in individuals with “*high plasticity”* has little observable effect whereas DBS withdrawal in conditions of “*low plasticity”* such as in chronic Parkinsonism or dystonia may result in status dystonicus (Trenado et al.).

Whereas the success of therapeutic interventions dominate all medical literature, defining the client population, instruments of intervention, definition and measurement of the successful outcome are the subject of endless debate about the value of case-report, cohort or randomized-control trial in supporting decision trees and guidelines. Often this begins by defining what we know and how we have come to such an understanding, first through the use of readily available diagnostic tools, then more complex, systems-based neurophysiological tools, followed by expert opinions, then inevitably, by clinical-neurophysiological ***supervised*** ***machine-learning*** algorithms that can shed light on how different dynamic systems respond to interventions such as DBS, allowing case-specific predictions of outcome. This might also be referred to as “*precision medicine”* that can identify a range of outcomes for individual clinical cases beyond the conventional statistical box and whisker plot of significant differences (McClelland and Lin).

Novel therapies also known as new ways of thinking about old problems are urgently needed and must be relevant to our understanding of neuroplasticity and neuromodulation.

Novel therapies include cognitive training strategies such as Cognitive Orientation to daily Occupational Performance (CO-OP) in childhood hyperkinetic movement disorders may be a more effective and low-cost means of achieving patient-selected functional goals and could be applied in health services worldwide, including resource-poor countries. CO-OP appears superior to repetitive practicing, especially preferable to practicing something in a way which is unhelpful (Gimeno et al.).

Another novel therapy could include “sub-threshold stimuli” such as “stochastic resonance (SR) sub-threshold mechanical noise stimulation” using sub-threshold vibrotactile noise stimulation of the wrist via a specialized wrist-watch. This may improve manual dexterity test scores in developmental coordination disorder (DCD). If repeatable and translatable to everyday life the smart watch of the future may deliver sub-threshold stimuli leading to motor improvements applicable to other motor disorders e.g., following neurological injuries or simply with motor decline of the elderly. Ultimately, we may all want one of these subthreshold stimulus systems!

Neuromodulation in its widest sense has no boundaries and consequently applications are potentially limitless and not necessarily costly.

The evolving concepts and tools elucidating neuroplasticity and neuromodulation, strongly support the case for further funding and training in the exciting fields of neuroplasticity, neuromodulation and neuro-rehabilitation.

## Author Contributions

The author confirms being the sole contributor of this work and has approved it for publication.

## Conflict of Interest

The author declares that the research was conducted in the absence of any commercial or financial relationships that could be construed as a potential conflict of interest.

## Publisher's Note

All claims expressed in this article are solely those of the authors and do not necessarily represent those of their affiliated organizations, or those of the publisher, the editors and the reviewers. Any product that may be evaluated in this article, or claim that may be made by its manufacturer, is not guaranteed or endorsed by the publisher.
